# Drug-likeness prediction of designed analogues of isoniazid standard targeting FabI enzyme regulation from P. falciparum

**DOI:** 10.6026/97320630015364

**Published:** 2019-05-15

**Authors:** Anil Kumar Pandey, Mohammad Haris Siddiqui, Rajiv Dutta

**Affiliations:** 1 Department of Bioengineering, Faculty of Engineering, Integral University, Lucknow - 226026, India; 2 Institute of Bio-Sciences and Technology, Shri Ramswaroop Memorial University, Lucknow Deva Road, Barabanki - 225003, India; 3 Department of Life Sciences, Faculty of Applied Sciences, Dr. K N Modi University, RIICO Industrial Area Phase-II, Newai, Tonk, Rajasthan - 304021. India

**Keywords:** Lipinski's rule, ChewDraw, FabI, isoniazid, analogues, malaria

## Abstract

Fatty acid biosynthesis enzymes (Fab enzyme) are important targets for anti-malarial drug development. The present study describes the toxicity screening of designed novel analogues which inhibit FabI enzyme regulation, a protein with multifunctional property. New analogues were prepared using ChemDraw Ultra 10 Software and converted into 3D PDB structure format for binding studies with FabI (PDB ID: 4IGE). Further Lipinski's rule of FIVE and ADMET profiling for toxicity prediction has been performed on the designed analogues. The result shows that ISN-23 is potential analogue exhibiting inhibition at the active site of FabI enzyme with good binding features.

## Background

Malaria is one of the most prominent tropical parasitic diseases 1. It has been revealed by the World Health Organization (WHO) that around 300�500 million sensitive clinical malarial cases every year and around 1 million deaths do occur every year 1. Malaria is mainly infected within the poorest populations in the World and it is widely spread in Africa, Asia, and in several South American countries. Malaria is mainly caused by four types of Plasmodium species, but Plasmodium falciparum is mainly important for the most serious and deadly form of the disease and is responsible for malaria-related deaths up to 90% in Africa 1. Medication of malaria is most and wide preference to the National Institutes of Health (NIH) and the significance approaches to the problem calls for multiple steps to tackle this world-wide problem. At present the therapeutic diagnose processes are concentrating in three main areas: (a) vaccine development, (b) drug development, and (c) pathogenesis. Within drug development there is a constant need to develop new drugs to overcome of existing ones for the treatment of malarial infections due to the severe problem of the growing resistance to known and present drugs. There is urgent requirement to identify and characterize the exclusive parasite biochemical pathways which may provide as targets for new drugs, to regulate the mode of action of existing and potential new drugs, and to elucidate possible mechanisms of resistance to existing drugs.

Available drugs respond to three classes of compounds: (1) aryl aminoalcohol compounds eg. quinine (2) antifolates dihydrofolate reductase inhibitors like pyrimethamine, and (3) Derivatives of artemisinin. Artemisinin was first isolated in 1970 by Chinese scientists from Artemisia annua 2,3. However, medication with only one drug is not acceptable and now there is a general agreement between scientists that synergistic effects of two drugs probably attempt to the best option for medication which reduces the risk of resistance. Examples of drug synergistic effects are the artemisinin-amodiaquine pair and the artemether-lumefantine 4. Fatty acids are universal in nature but marine organisms, such as particular sponges, have provided a platform for some of the most interesting varieties based on structure. Most of among these marine fatty acids comes from unusual biosynthetic pathways and excellent reviews have noticed in recent years as the fatty acid varied structure types present in these organisms, their main role in membranes, and their biogenesis processes 5-8. However, very short is known, or has been revealed, that biomedical potential of these remarkable sponge fatty acids; in particular as to what differences exist in their bioavailability in comparison to reported for the more common fatty acids. Due to present activity in research, we are now able to begin in learning more as to the potential of these marine compounds to conflict of these infectious diseases such as malaria, tuberculosis, and fungal infections. Malaria chemotherapy is an area that is in continuous growth and revision due to the limited number of drugs presently available, the severe side effects of available drugs, and the continuous development of resistance developed by the parasite to some of these drugs 9. P. falciparum, the malaria parasite of the phylum Apicomplexa which contains an apicoplast, an organelle that originally arise from a cyanobacterium through a process of secondary endosymbiotic and thus shows two membranes 10. The malaria parasite which contains apicoplast is indispensable for several vital metabolic processes for the parasite do occur at this site. Among these the main processes are isoprene biosynthesis, haem biosynthesis, and fatty acid biosynthesis take place. Higher eukaryotes normally use a type I fatty acid synthase (FASI) system, where each fatty acid biosynthetic step is catalyzed by a single protein with multiple domains. On the other hand, in the apicoplast a type II fatty acid synthase (FASII) system is operative, where each fatty acid biosynthetic pathway is carried out towards a discrete enzyme encoded by different gene 11. In human type II FAS system is absent since we are eukaryotic in nature but is common in bacteria and algae 12. When the parasite is invading a host it needs to hide itself by creating a parasitophorous vacuole, which imparts a protection of the host immune system. The parasite needs to make its own fatty acids in this process for de novo so as to form its membrane expanded. In P. falciparum the principal membrane fatty acids are decanoic acid (10:0), lauric acid (12:0), and myristic acid (14:0). There are several enzymes responsible for the biosynthesis of fatty acids in P. falciparum which are harmful for human during erythrocytic phase of incubation. Hence, the incorporation of these several enzymes can be inhibited by drugs. Some known drugs are isoniazid (which inhibits FabI), and thiolactomycin and derivatives (which inhibit FabB and FabH) 13, 14.

The anti-malarial effect of fatty acids has propelled towards deliberation in the past but the realization that fatty acids themselves might inhibit the fatty acid biosynthetic machinery of the parasite P. falciparum has only been presently examined to make strategy towards combating of parasite. It is known that that antimalarial property of n3 and n6 fatty acids which were polyunsaturated in nature postulates the in-vitro invasion of intra erythrocytic forms of P. falciparum 15. The methyl esters of the fatty acids were reported to be as potent as the free acids in killing the parasite. The binding of the fatty acids to albumin in vivo was also discussed as unlikely to inhibit the anti-malarial effect of the polyunsaturated fatty acids 15.

## Methodology

### Target protein structure:

The structures of enzymes (FabI) involved in Plasmodium falciparum regulation was obtained from Protein Data Bank (PDB ID: 4IGE).

### Prediction of active sites:

Meta pocket 2.0 Finder was used for several separate procedures to perform active/ binding site prediction (Table 1). To minimize the volume of the box (pocket) enclosing the protein is carried out by generating their coordinates. Every probe coordinates are then clustered according to their spatial proximity, and the full interaction to their energies of probes. This leads to connects all adjacent sites but not on the diagonals of the cube. The probe clusters were ranked according to their total interaction energies, with the most remarkable being identified as the first predicted binding site. The variables for estimation of site volume and identification of proteins or enzymes were predicted by Meta pocket.

### Preparation of compounds/analogues:

The 2D-structure of Isoniazid and its 23 analogues were designed by using ACD lab software extension ChemDraw in MDL .mol format.

### Drug-likeliness prediction for isoniazid analogues:

The Lipinski rule of FIVE predicts the pharmacological, biological and ADME (absorption, distribution, metabolism and excretion) exercise of the particular compound and also predicting its potentiality to an orally active drug in humans 16.

### ADMET analysis for isoniazid analogues:

Using Pre ADMET online server 20 the pharmacokinetics criteria like Adsorption, Distribution, Metabolism, Excretion and Toxicology (ADME/T) was performed. The properties like Human Intestinal Absorption (% HIA), Caco-2 permeability, MDCK cell Permeability, Skin Permeability, Blood Brain Barrier Penetration and Carcinogenicity all these parameters were deliberated.

## Results and Discussion

Results show that Isoniazid drug inhibit the fabI enzyme regulation during the erythrocytic phase of parasitic incubation. Hence, the active site of FabI enzyme was predicted. Thus, different amino acids residues of the enzymes were found out using meta-pocket 2.0 Finder (Table 1). The residues with potential/active binding sites in FabI (PDB ID: 4IGE) are: Gly104,Ile105, Gly106, Gly110, Tyr111, Trp131, Phe167, Asp168, Ala169, Ser170, Ser215, Leu216, Ala217, Asn218, Leu265, Thr266, Tyr267, Lys285, Ala312, Gly313, Pro314, Leu315, Ser317, Ala319, Ala320, Ile369. The designed analogues (2D structure) were converted into 3D-structures using Discovery Studio 2.5 (Table 2). Further, ADME as well as druglikeness were evaluated for the designed analogues. Potential compounds then further examined for their pharmacokinetics properties, metabolism and potential toxicity. Thus, combinatorial chemistry and high throughput ADME screening were completed. ADMET prediction of analogues was completed using the PreADMET online tool 20 as given in Table 3 and Table 4. Druglikeliness of the 23 designed compounds was assessed using the Lipinski's rule of 5 17, 18 followed by ADMET evaluation (Table 5). Results show that ISN-23 analogue have the best binding features with FabI enzyme for further in vitro and in vivo consideration.

## Conclusion

Various designed analogues or compounds are associated with known anti-malarial drugs (isoniazid standard) by clocking FabI enzyme in the treatment of malaria caused by P. falciparum. We document the predicted binding of 23 designed analogues with the FabI enzyme and show that ISN-23 analogue have the best binding features with FabI enzyme for further in vitro and in vivo consideration.

## Conflict of Interest

Authors declare no conflict of interest.

## Figures and Tables

**Table 1:** The potential binding sites/active site in FabI (4IGE) using Meta Pocket

List of Binding Sites
RESI	GLY104	ILE105	GLY 106	ASP107	ASN109
RESI	GLY110	TYR111	GLY112	TRP113	SER215
RESI	LEU265	LEU216	ALA217	ALA217	SER317
RESI	HIS214	LYS285	SER264	ASN218	ALA219
RESI	VAL222	ALA322	TYR277	ALA320	MET281
RESI	ILE323	TYR267	PHE368	ILE369	THR266
RESI	PRO314	ALA372	ASN141	LYS146	PHE147
RESI	ILE137	PHE138	ARG318	VAL134	THR108
RESI	ASP150	TRP131	GLY129	PHE167	LYS240
RESI	ALA169	LEU315	ASP168	SER170	GLY313
RESI	LYS220	ALA312	GLN223	THR181	ASN184
RESI	TYR187	GLU180	ASP178	PRO133	PHE171
RESI	ASP236	LYS316	THR321	SER239	SER244
RESI	SER241	VAL274	LEU288	SER311	

**Table 2:** Leads/analogues designed/constructed by taking isoniazid as a standard

Sr. No.	Canonical SMILE Version
1	C1=CN=CC=C1C(=O)NN
2	C1([H])=C(C(N=N)=S)C([H])=C([H])N=C1[H]
3	C1([H])=C(C(N=N)=S)C([H])=C([H])P=C1[H]
4	C1([H])=C(C(N=N)=S)C([H])=C([H])P=C1O
5	C1(O)=C(C(N=N)=S)C([H])=C([H])P=C1O
6	C1(O)=C(C(N=N)=S)C(O)=C([H])P=C1O
7	C1(O)=C(C(N=N)=S)C(O)=C(O)P=C1O
8	C1([H])=C(C(N=N)=O)C([H])=C(P[H])N=C1O
9	C1(O)=C(C(N=N)=O)C([H])=C([H])N=C1O
10	C1(O)=C(C(N=N)=O)C(O)=C([H])N=C1O
11	C1(O)=C(C(N=N)=O)C(O)=C(O)N=C1O
12	C1([H])=C(C(N=N)=[F+])C([H])=C([H])N=C1[H]
13	C1([H])=C(C(N=N)=[Cl+])C([H])=C([H])N=C1[H]
14	C1([H])=C(C(N=N)=[Br+])C([H])=C([H])N=C1[H]
15	C1([H])=C(C(N=N)=[I+])C([H])=C([H])N=C1[H]
16	C1(O)=C(C(N=N)=[F+])C(O)=C(O)N=C1O
17	C1(O)=C(C(N=N)=[Cl+])C(O)=C(O)N=C1O
18	C1(O)=C(C(N=N)=[Br+])C(O)=C(O)N=C1O
19	C1(O)=C(C(N=N)=[I+])C(O)=C(O)N=C1O
20	C1([H])=C(C(N=N)=[F+])C(O)=C(O)N=C1[H]
21	C1([H])=C(C(N=N)=[Cl+])C(O)=C(O)N=C1[H]
22	C1([H])=C(C(N=N)=[Br+])C(O)=C(O)N=C1[H]
23	C1NC(C(C(C1)/C(=N\N)/I)O)O
24	NC(=O)C1CCNCC1

**Table 3:** Pharmacokinetic studies to measure the drug concentrations in blood or plasma

Sr. No.	ADME Properties	Activity Range
1	Human intestinal absorption (HIA)	Poorly- 0 ~20%
		Moderate- 20 ~70%
		High- 70 ~100%
2	Blood brain barrier (BBB)	CNS active compounds (+); >1
		CNS inactive compounds (-); < 1
3	Madin-Darby canine kidney (MDCK)	Lower- < 25
	cell permeability	Moderate- 25 ~500
		Higher- > 500
4	Heterogenous human epithelial	Lower- < 4
	colorectal adenocarcinoma (Caco2)	Moderate- 4 ∼70
	cell permeability	Higher- < 70
5	Plasma protein Binding (% PBP)	Chemicals strongly bound > 90%
		Chemicals weakly bound < 90%

**Table 4:** ADMET prediction of novel designed Analogues with compare to parent compound

Sr. No.	Lead Name/Symbol			ADME Properties			
							
		Caco2	MDCK	SP	HIA	BBB	Toxicity
							(M/C)
1	Isoniazid	9.76	0.53	-5.31	82.42	0.09	-/-
	(Standard)						
2	ISN-1	19.764	5.63168	-2.83253	90.82	0.24	+/-
3	ISN-2	19.59	4.11	-4.01	73.6	0.15	*OR
4	ISN-3	16.53	2.89	-4.15	52.13	0.09	*OR
5	ISN-4	15.11	1.19	-4.15	30.38	0.06	*OR
6	ISN-5	12.02	0.97	-4	15.73	0.05	*OR
7	ISN-6	6.48	0.71	-3.88	7.91	0.04	*OR
8	ISN-7	5.69	33.34	-4.33	54.55	0.54	±
9	ISN-8	1.14	2.59	-4.92	33.33	0.18	±
10	ISN-9	0.54	0.94	-5.07	16.53	0.09	±
11	ISN-10	0.53	0.71	-5.08	6.95	0.06	±
12	ISN-11	20.05	3.27	-3.02	94.14	0.49	+/+
13	ISN-12	19.71	4.2	-2.83	95.64	0.55	+/-
14	ISN-13	19.52	0.46	-2.8	95.81	0.58	+/+
15	ISN-14	19.47	0.51	-2.81	96.46	0.6	+/+
16	ISN-15	7.56	2.25	-4.85	26.82	0.1	±
17	ISN-16	13.04	1.48	-4.76	41.83	0.12	+/+
18	ISN-17	15.37	0.59	-4.71	53.24	0.12	+/+
19	ISN-18	15.96	0.5	-4.68	84.63	0.13	+/+
20	ISN-19	16.51	2.61	-4.44	71.78	0.31	±
21	ISN-20	18.55	2.75	-4.2	82.26	0.32	-/-
22	ISN-21	19.2	0.76	-4.08	86.42	0.33	±
23	ISN-22	19.35	0.5	-4.05	92.84	0.34	±
24	ISN-23	20.36	2.78	-3.72	92.94	0.33	-/-

**Table 5:** Drug-Likeliness prediction of designed analogues and standard drug

Sr. No.	Analogues	MW	HBA	HBD	Log P
1	Isoniazid (standard)	137.06	3	3	-1.03
2	ISN 1	151.02	4	1	0.91
3	ISN 2	167.99	3	1	1.47
4	ISN 3	183.99	4	2	1.18
5	ISN 4	199.98	5	3	0.68
6	ISN 5	215.98	6	4	0.3
7	ISN 6	231.97	7	5	-0.12
8	ISN 7	151.04	5	2	0.35
9	ISN 8	167.03	6	3	-0.15
10	ISN 9	183.03	7	4	-0.53
11	ISN 10	199.02	8	5	-0.64
12	ISN 11	138.05	3	1	0.99
13	ISN 12	154.02	3	1	1.01
14	ISN 13	197.97	3	1	1.21
15	ISN 14	245.95	3	1	1.25
16	ISN 15	202.03	7	5	0
17	ISN 16	218	7	5	0.02
18	ISN 17	261.95	7	5	0.22
19	ISN 18	309.93	7	5	0.26
20	ISN 19	170.04	5	3	0.49
21	ISN 20	186.01	5	3	0.51
22	ISN21	229.96	5	3	0.72
23	ISN22	285	4	5	-0.9
24	ISN23	128.092	3	-0.88
